# How to Analyze (Intentional) Consciousness in Terms of Meta-Belief and Temporal Awareness

**DOI:** 10.3389/fpsyg.2018.01628

**Published:** 2018-09-07

**Authors:** Christian Beyer

**Affiliations:** Philosophisches Seminar, Georg-August-Universität Göttingen, Göttingen, Germany

**Keywords:** consciousness, intentionality, metarepresentation, unity of consciousness, temporal awareness, Husserl

## Abstract

The paper presents and defends a metadoxastic view on (intentional) consciousness that is novel in four respects: (1) It is motivated both by Husserl’s dynamic approach, which looks upon mental acts as momentary components of certain cognitive structures – “dynamic intentional structures” – in which one and the same object is intended throughout a period of time (during which the subject’s cognitive perspective upon that object is constantly changing) and by his conception of consciousness in terms of internal time-consciousness (temporal awareness). (2) It combines a dispositionalist higher-order judgment theory about the structure of (intentional) consciousness with the claim that the contents of these judgments are such that they can be expressed by essentially indexical sentences containing the temporal indexical “now,” thus accommodating the basic role of internal time-consciousness. (3) It is immune against the “objection from lack of mental concepts” raised, e.g., by Dretske against any higher-order representation theory, as it employs counterfactuals in the framework of a disjunctive account of (intentional) consciousness. (4) It explains the unity of consciousness at a time as well as across time.

## 1. The Metadoxastic View and Its Phenomenological Motivation (Husserl)

We are equipped with intentional consciousness. In other words: we have the ability to refer to something consciously. Following Husserl, I assume that, at least phenomenologically, this ability depends on what might be called, in a more recent terminology, our *cognitive-dynamic* capacities: we seem to be able to keep track of objects (including states of affairs) across time and this ability seems to enable us both to have particular objects in mind at a given time and to talk about those objects.^1^ As Husserl observed, any intentional experience of this sort occurs in the framework of a transtemporal structure of (momentary) intentional states all of which purport to refer to one and the same object. (They belong, as Husserl puts it, to the same determinable X. The transtemporal structure held together by such a determinable X could be called a *dynamic intentional structure*.) Thus, for instance, I have a lot of expectations, or anticipations, about the way this particular table will, or may, appear to me if I walk around and observe *that selfsame table* from different perspectives. This presupposes that I have an idea of what it means for me to keep in view a particular thing throughout a period of time. Expectations such as this are essentially involved even if I perceive something as a table more or less statically (at a glance): I cannot see an object as *a table*, unless I expect it to display various sides and aspects, to be usable in a number of typical ways, etc. On closer inspection, these expectations turn out to be *higher-order* intentional states, i.e., *meta* representations: notably conscious or unconscious anticipations about the kinds of intentional experiences I will (or may) undergo if I perceive the table from such-and-such a perspective as well as about the intentions I will (or may) have as an agent regarding that table under such-and-such circumstances, and so on.^2^

Now if higher-order beliefs about the *future* build an essential prerequisite, in the way just illustrated, for intentional consciousness – as Husserl argues, focussing on his paradigm example of intentionality, i.e., conscious perception – then it is plausible to assume that this consciousness goes hand in hand with higher-order beliefs about the *present* (and, for that matter, about the past) as well; after all, anticipations about future experience do not drop from the sky. This is where the metadoxastic view on intentional consciousness comes in. On this view, intentional consciousness is normally accompanied by present-tense higher-order belief, on the grounds that (as a first approximation) it is the presence of such higher-order beliefs (and the particular temporal sensations motivating them; see below) that marks the difference between *conscious* intentional states and unconscious ones. Thus:

The metadoxastic view on intentional consciousness (1^st^ approximation)

A given intentional state is conscious (is an intentional *experience*) iff the subject being in that state believes him- or herself to be in that state, where this meta-belief is based upon (is caused or motivated by) that lower-order intentional state.

The right-hand side of this bi-conditional is supposed to make explicit, or explicate, our implicit notion of intentional consciousness. This explication orients itself by what we would, or should, say, in the light of our philosophically and scientifically informed (intersubjective) belief-system, about the nature and structure of consciousness; where *intentional* (i.e., object-directed) consciousness is regarded as the paradigmatic case, the conceptual explication of which is the core of an understanding of consciousness in general. On the metadoxastic view, higher-order beliefs are part of the *structure* of intentional consciousness. The metadoxastic view results from an (empirically informed) intentional analysis – an analysis of the “essential” (Husserl) or “logical” (Searle) properties and the corresponding structural features of intentional consciousness, performed from the first-person point of view. Thus, the following considerations do not concern the *sub*-personal mechanisms underlying consciousness, although hypotheses about these mechanisms, to the extent that they are justified, are relevant for a well-informed, coherent conception of intentional awareness. Nor does the metadoxastic view amount to a *reductive* thesis (even concerning the *personal* level) in the sense of the assertion of a “nothing-but” relation (compare and contrast “genes are nothing but DNA molecules;” cf. Searle, 1992, p. 113). Rather, this view makes explicit conceptual relations in which the notion of intentional consciousness, conceived from the first-person point of view, stands to other notions belonging to the theory of intentionality. In particular, it assumes that the essential features of intentional consciousness can be brought to light by a conceptual analysis, from the first-person perspective, of the relations of (pre-predicative or predicative) motivation and mereological foundation (ontological dependence) obtaining between intentional states and their dependent parts in the case of intentional consciousness. Some of these essential features (in particular, the feature of temporal awareness) turn out to be more fundamental than others in the sense that those other features (in particular, the relevant meta-beliefs) are motivated by and founded in them (see below). However, both of these features are held to contribute, at different levels of mereological foundation, to the essential structure of intentional consciousness.

On the metadoxastic view, our notion of, say, my consciously perceiving the table over there as a table involves the idea of my *believing* myself to perceive that table under such-and-such aspects now; which implies, in turn, that I expect that selfsame table to present itself to me in certain ways, e.g., in a characteristic series of perceptual “adumbrations” (Husserl), if I walk around and keep observing the table (see above). Note that apart from the notion of an intentional state of such-and-such a sort (here: of a perception displaying a particular content), the (propositional) content of the relevant meta-belief contains *indexical* concepts – concepts whose reference systematically depends upon the context of thought or utterance: notably, the respective concepts of the self (“myself”) and the present (“now”). It is for this reason that, as David Rosenthal has observed, we can report our conscious intentional states in the following style: “*I am presently* seeing a so-and-so,” “*I now* see that such-and-such is the case.”^3^ The contents of these subjective reports coincide with the contents of the indexical meta-beliefs which (in the case of normal adults; see below) form the top of the intentional structure of *conscious* mental states.

So far, I have sketched a particular conception of the structure of intentional consciousness. I have avoided the claim that the indexical meta-beliefs forming the top of this – itself intentional – structure *constitute the true nature* of consciousness, in the sense that consciousness is “nothing but” appropriately caused or motivated indexical meta-belief. This is because I agree with Husserl and his interpreter Dan Zahavi that consciousness does not derive from (is neither grounded in nor reducible to) higher-order representation. Rather, it is the time-consciousness inherent to lived experiences that accounts for the fact that they flow in the stream of consciousness – and that at the same time makes them available (if the required conceptual abilities are in place; see below) for what Husserl calls “reflexive [*reflektives/reflexives*] self-awareness,” i.e., for the Cartesian “cogito,” such as the meta-judgment “I am observing a house” (conceived along the lines of the methodological solipsism Husserl adopts in the context of his analysis of the constitution of consciousness). When we undergo a given experience in the ordinary, self-forgotten way it is “latent,” says Husserl; it only becomes “patent” by the “occurrence of an Ego reflecting upon it,” which is in turn latent.^4^ In other words, when reflexive self-awareness occurs, it is itself available (accessible) for higher-order judgments but does not require their occurence either in order to occur.

Husserl stresses that “self-forgotten” or “latent” first-order consciousness is “prior” to its becoming “patent” by reflexive self-awareness (introspective judgment) and that one could “speak of a *latent Ego”* in connection with first-order consciousness.^5^ This lends support to Zahavi’s claim that on Husserl’s view consciousness is marked by “pre-reflexive self-awareness” (to use a term Zahavi adapts from Sartre)^6^ and that Husserl does not endorse an actualist higher-order thought theory of consciousness (according to which consciousness must always be accompanied, and represented, by reflexive self-awareness; see section 2 below). As the “latent”/manifest” terminology indicates, though, there is an internal connection between pre-reflexive and reflexive self-awareness. I propose to spell this out in terms of the indexical metadoxastic view.^7^ (Note in this context that Husserl regards beliefs as personal “habitualities” – and that his notions of habituality and latency are closely linked.)^8^ It is at this point that Zahavi rejects my reconstruction of Husserl’s conception of consciousness. He asks:

“How can one at the same time argue that inner consciousness is what motivates and founds the higher-order disposition and still defend a capacity-based explanation of consciousness, i.e., an account that our mental states are conscious if and only if we have the capacity to judge that we are having them?”^9^

My answer is twofold. First, I do not subscribe to, nor do I ascribe to Husserl, an explanation of consciousness in terms of higher-order judgmental dispositions, in the sense of a reductive thesis about consciousness. I do *not* claim that on Husserl’s view “a mental state is conscious *because* it possesses a dispositional metarepresentation.”^10^ In the article Zahavi refers to, I make it clear that the dispositional higher-order view I ascribe to Husserl concerns “the structural features” of the elements of the stream of consciousness, as opposed to a thesis about the “intrinsic nature of consciousness.”^11^

Having said this, I should stress that Husserl *does* regard the relevant top-structure of dispositional metarepresentation as belonging to the nature of consciousness – but at a less fundamental level. This brings me to the second part of my answer: Zahavi is right that the relevant meta-beliefs ought to be conceived as mental abilities or *capacities* in the context of my interpretation of Husserl. In fact, there are a number of passages cited in both my and Zahavi’s articles^12^ in which Husserl says that the constant “possibility” or “ability” to perform corresponding acts of reflexive self-awareness belongs to the regional *essence* of consciousness, precisely because it is marked by pre-reflexive self-awareness. Thus, he states that consciousness is by its very nature always reflexively *available*.^13^ Note that Husserl only knows two kinds of possibility: mere logical (or “ideal”) possibility, on the one hand, and real possibility, on the other;^14^ where the real possibility to acquire knowledge regarding a contingent object A “requires” an “epistemic subject” which “has the practical possibility (or the practical ability) to experience A and acquire knowledge regarding it.”^15^ Accordingly, the real possibility to acquire knowledge by reflexive self-awareness requires the epistemic *capacity* to make a corresponding higher-order judgment. This is just what the metadoxastic view of consciousness (conceived as a view on the intentional structure of consciousness) would lead one to expect. It implies that the structure of (normal adult; see section 3 below) intentional consciousness includes a level of meta-belief, in the sense that the respective subject of consciousness *can* judge him- or herself to be in a particular intentional state right now. This may sound like a rather weak claim, but the underlying sense of “can” is quite strong: it refers to the *real* possibility – the personal disposition – to make a corresponding introspective judgment. In order for this real possibility to obtain, the possession of the concepts required to make such a judgment is not sufficient, even if there is appropriate temporal awareness. Rather, the corresponding disposition (and its mental manifestation, if any) must be mereologically founded in and motivated by that temporal awareness.

On my view, it is in this sense – reflexive availability, conceived as a higher-order epistemic (and thus representational) capacity or disposition – that the underlying internal time-consciousness (à la Husserl) may be described as “pre-reflexive self-awareness.” True, Husserl would argue that “a mental state possesses a dispositional metarepresentation because it is conscious,”^16^ and not vice versa. But he does hold that the dispositional reflexive self-awareness (reflexive availability as a real possibility) therefore belongs to the essence of consciousness (see above); and it seems to me that there is no point in describing the underlying time-consciousness as *pre*-reflexive self-awareness without regarding it as embedded in an intentional structure also comprising, at a less fundamental level, a corresponding capacity for *reflexive* self-awareness (see below).^17^ It is in this sense that I have claimed that “[w]ithout the corresponding higher-order dispositions to judge, ‘inner consciousness’ would remain blind, and it would hardly be appropriate to designate it as ‘pre-reflexive self-consciousness’.”^18^

To highlight further the motivating function of internal time-consciousness, and its relation to the indexical concepts involved in the intentional content of the relevant meta-beliefs, I shall add a few details of Husserl’s analysis of reflexive self-awareness, i.e., introspective judgment (where the “latent Ego” becomes “patent”). Whenever such self-awareness occurs, it is founded, on his view, in “higher-order perception” (Husserl, 1950-, Hua VIII, p. 88), i.e., “inner perception” of the lived experience reflexively grasped. This inner perception is founded both in a *retention*, which allows it to intuitively grasp and immediately retain (“*zurückgreifendes Nachgewahren*”) the directly preceding phase of the perceived lived experience, and in an *original impression*, which helps the current phase of the lived experience to present itself (see also section 4 below). That the ‘inner’ perception is founded in this internal time-consciousness (original impression plus retention) means that it cannot exist without the latter, as a matter of an essential law (concerning the structure of consciousness). Unlike in the case of outer perception, the perceived object does not “adumbrate itself” perspectively in ‘inner’ perception^19^ but presents itself as it intrinsically is qua lived experience, notably in its essential character as a conscious experience, the living present.

Note that this essential character consists in the lived experience’s being sensed, albeit not in the form of a perspectival adumbration, as “now” (original impression) and then “just a moment ago” (retention). On Husserl’s view, we are not dealing with an *intentional* experience here but rather with a “sensation (*Empfindung*)” and its immediate “resonance (*Nachklang*)” (Husserl, 1950-, Hua X, pp. 146f), as opposed to an intentional “positing” (an ‘inner’ perception) founded in such a sensation-*cum*-resonance (cf. Husserl, 1950-, Hua X, pp. 126f; also cf. Husserl, 1950-, Hua X, pp. 295f). This type of temporal sensing, consisting of an original “impression (*Impression*)” and a non-representational “reproduction (*Reproduktion*)” (retentional modification) of the immediate subjective past (Husserl, 1950-, Hua X, pp. 295f), has the effect that consciousness flows in a steady stream, so to speak – it accounts for its peculiar flow character. What I experience “right now” is immediately transformed into what I experienced “just a moment ago,” by means of a retention that keeps alive what has been experienced just before. (Notice that Husserl regards both original impressions and their immediate retentional modifications as “immune to any reasonable doubt,” thereby commiting himself to the view that the immediate subjective past thus sensed undoubtedly exists; cf. Husserl, 1950-, Hua X, pp. 343f.) “Before,” “afterward,” “at the same time” – this is how consciousness is always already structured, as it is automatically founded in according temporal sensings (original impression plus retention), not to be confused with corresponding ‘inner’ perceptions or acts of reflexive self-awareness based upon such perceptions. Internal time-consciousness is thus a mere sensing that is constitutive, in the way just indicated, of the flow character of lived experience, and it *motivates* corresponding acts of ‘inner’ perception and reflexive self-awareness. However, this motivation can only occur if the respective subject already has the notions of self (“I”), present (“now”) and the required mental concepts (if any) in his or her conceptual repertoire. A small child or non-human animal (still) lacking those concepts could perhaps undergo lived experiences (be in conscious mental states), thanks to the internal time-consciousness built into these experiences, but here there is no “latent Ego” that could become “patent.” It is somewhat doubtful whether Husserl can allow for this possibility, as he regards reflexive availability as an essential feature of consciousness (see above); but perhaps he means “reflexive availability provided the subject possesses the required concepts.” In any case, it is only when internal time-consciousness can actually fulfill the described motivating function that it functions as “pre-reflexive self-awareness.” It needs an intentional super-structure for this purpose. (Without such a super-structure, it merely functions as *pre-*pre-reflexive self-awareness, provided that the animal in question has the learning potential required to acquire the relevant concepts.) I propose to conceive of this super-structure as the capacity for reflexive self-awareness and thus to embed Husserl’s conception of (normal adult intentional) consciousness in a version of the metadoxastic view that takes into account the conceptual repertoire required for such self-awareness.

In what follows I will develop further this *metadoxastic indexical view (on the structure of intentional consciousness)* and defend it against, or amend it in the light of, some objections. In this way, the theoretical consequences – and indeed advantages – of embedding Husserl’s conception in this view will become clear. But first, let us get clear about what we take to be, on the one hand, the ontological status of the relevant indexical meta-beliefs and, on the other hand, their (normal) function with respect to intentional consciousness. It is helpful to perform this task in connection with an objection that can be raised against another higher-order representation theory of consciousness, namely the *actualist higher-order judgment (thought) theory*.

## 2. The Actualist Higher-Order Judgment View

This theory holds that in order for me to consciously perceive, say, a table, I must make a meta-judgment, to the effect that I am perceiving something as a table. (Where judgments are to be conceived of as momentary belief states that are activated in the course of a cognitive process, such as an observation, rather than as momentary states of intentional consciousness.) This idea can be generalized as follows:

Actualist higher-order judgment (thought) theory

A given subject is in a conscious intentional state (undergoes an intentional experience) iff the subject judges that he himself, or she herself,^20^ is in that state; where this meta-judgment is based upon (is caused or motivated by) that lower-order intentional state.

The relevant objection [objection #1] derives from something like folk-psychology-*cum*-phenomenology. On all accounts, there seems to be an obvious difference between conscious and unconscious perception. Consider the following example by David Armstrong. A person who has been driving his car for some hours suddenly “comes to” and realizes that he has not been paying attention to his driving activity for some time. Yet, he surely must have perceived the street, albeit unconsciously; for otherwise he would have built an accident.^21^ But now, even as an *attentive* driver I do not permanently think by myself “I am now seeing the street” or the like. Nevertheless, I am undergoing perceptual *experiences*. Hence, the objection ends, the actualist higher order judgment theory is folk-psychologically and phenomenologically implausible as well.

## 3. The Indexical Metadoxastic View: a Defense

The indexical meta*doxastic* view I want to defend is immune to this objection. For it conceives of the meta-beliefs associated with intentional consciousness as mere judgmental *dispositions*.^22^ And the attentive car-driver does not have to assert, *in foro interno*, something like “I now see the street” in order to be *disposed* to make the meta-judgment that can be given voice to by this sentence.^23^

There is, however, another objection that can be raised both against the actualist higher-order judgment theory and, *mutatis mutandis*, against the metadoxastic view [objection #2]. According to the metadoxastic view, a given subject is in an intentional state of such-and-such a sort only if the subject believes him- or herself to be in a state such as this. Now it looks like in order to have this meta-belief the subject must possess the *concept* of an intentional state of the relevant sort. After all, you cannot be disposed to judge that you now believe (/perceive/hope/desire/...) thus-and-so, unless you have at your disposal at least a rudimentary “I”-concept as well as the concept of belief (/perception/hope/...).

Let us focus on the concept of belief. As developmental psychology has taught us, children acquire the folk-psychological concept of belief, according to which beliefs may be *false*,^24^ only around the age of four.^25^ In fact, smaller children may not even have a *rudimentary* concept of belief. However, this poses a serious problem for the metadoxastic view. After all, young children do seem to have intentional consciousness.^26^ E.g., they do seem to be able to have conscious beliefs or to make (conscious) judgments. But if the metadoxastic view, as it stands, were correct, they could not. After all, they do not yet know what it means to believe that something is the case.

Notice, however, that from a certain age children do at least possess the *learning potential* required for the acquisition of the concept of belief. Therefore, I think that the following type of counterfactual can hold true of small children as well: “*If the child already had the concept of belief in his or her repertoire*, then he or she would be disposed to judge that he or she him- or herself now believes that thus-and-so.”^27^ And it seems to me that if a counterfactual such as this is indeed true in virtue of a momentary belief state of the child’s, we can plausibly ascribe to him a *conscious* belief state or a judgment. For instance, if 3-year old Anna notes, while looking out of the window: “It’s raining,” then she is plainly consciously *aware* of the fact that it is raining – she makes the corresponding judgment. For if she already had the concept of belief in her repertoire, she could just as well make, with the true ring of conviction, the following assertion: “*I now see* that it’s raining.”^28^ What is it, though, that *makes true* the relevant counterfactual? I think that its truth can be explained in two different ways: (1) in phenomenological terms; (2) in terms of the Theory of Evolution. (1) If Anna already had the concept of perceptual belief (and the concepts of self and present), she would be disposed to judge herself to be presently seeing that it is raining. This disposition (or epistemic capacity) obtains because of the inner time-consciousness in which Anna senses the flow character of her perception, which time-consciousness turns into pre-reflexive self-awareness (and thus motivates the belief to be expressed by “I now see that it’s raining”) once Anna has the required concepts (see section 1 above). (2) The evolutionary explanation is along the following lines: conscious belief-states have the biological *function* to cause the respective subject to be disposed to make appropriate meta-judgments under specific circumstances, so that the subject gets into a position, e.g., to intentionally deceive others regarding his lower-order belief states (see fn. 28) or to solve complex problems; where in the case of small children conscious belief states do not yet function the way they ought to.^29^ In the case of *very* young children (babies), belief states such as this probably do not function at all yet, so that they are still lacking any judgmental capacity whatsoever. The question whether this supposition is true can only be answered by developmental psychology, though. The same goes for the question of whether the Great Apes and other higher animals have conscious belief states at their disposal. In any case, it seems clear that lower animals like insects cannot intentionally deceive others regarding what they perceive or believe and that they cannot solve complex problems. Furthermore, there is no reason to suppose that they have the learning potential required for the acquisition of the notions of self and present. It is therefore unlikely that their perceptual system involves temporal sensations whose motivating function would precisely consist in their giving rise to the application of such concepts in reflexive self-awareness. Rather, it is likely that they lack internal time-consciousness (even in the form of *pre*-pre-reflexive self-awareness; see first section, last but one paragraph) altogether. Thus, I plead for a version of the metadoxastic view that makes recourse to counterfactuals. As a first approximation, my proposal runs:

Dispositionalist indexical higher-order judgment theory (version #1)

A given subject is in a conscious intentional state (undergoes an intentional experience) iff the subject *either* believes that he himself, or she herself, is in that state *or would* believe so iff he or she already possessed the concept of an intentional state such as this; where this meta-belief is, or would be, based upon (be caused or motivated by) that lower-order intentional state.

However, this proposal needs some further refinement. It should take into account the following possibility [objection #3]: the visual field of a person with *blindsight* contains a blind area (the *scotoma*), but the person can nonetheless unconsciously perceive an object presented to him or her in this area; and it is well possible that he or she is disposed, on the basis of both this unconscious perception and background-information about his state as well about as the experimental setting, to *inferentially* arrive at the meta-judgment that he himself is perceiving the object in question.^30^ In order to exclude this kind of case, which is after all not a case of *conscious* perception, the indexical metadoxastic view should make recourse to the notion of a *non-inferential* meta-belief, as follows:

Dispositionalist indexical higher-order judgment theory (version #2)

A given subject is in a conscious intentional state (undergoes an intentional experience) iff the subject *either* non-inferentially believes that he himself, or she herself, is in that state *or would* non-inferentially believe so if he already possessed the concept of an intentional state such as this; where this meta-belief is, or would be, based upon (be caused or motivated by) that lower-order intentional state.

The person with blindsight may believe him- or herself to perceive an object in the blind area, but then he or she will have arrived at this meta-belief inferentially (on the basis of the relevant background-information), so the present theory of consciousness does not apply. Note that “non-inferential belief” is used as a term of *personal*-level psychology here. Thus, hypotheses about sub-personal, unconscious inferential processes underlying perceptual consciousness remain unaffected. Nor does the recourse to non-inferential belief exclude fictitious examples in which blindsight subjects simply find themselves believing that they are perceiving an object, due to a non-inferential belief-forming mechanism (say, because God has put the right belief in their head).^31^ It merely excludes cases where the meta-belief in question is arrived at by a personal-level inference referring to appropriate background information.

An integral element of this theory, which I will return to in section 4 (also see section 1 above), is the phenomenological claim that intentional consciousness is always founded in a form of time-consciousness: if I take myself to be in such-and-such an intentional state *now*, then it seems that phenomenologically I must be aware of *the present moment* that the temporal demonstrative refers to (although it does not seem necessary for me to be explicitly aware of either my “self” or the intentional state in question, which is to say that the relevant meta-belief need not be conscious). For if the present were not somehow “present” to me, I would at best be mentally absent now and could hardly be said to undergo an intentional *experience*. Note that in this case the present moment must have been *in the future for me* just a moment ago, and that it will be *in the past for me* in just a moment. It is in this sense that the present moment, just like a momentary phase of a *movement* that is being observed, is part of a temporal series which is continuously “constituted” by time-consciousness and which is thereby permanently in a state of flux *qua* (the indexical aspects) future, present and past.^32^

The fact that this temporal series is constantly “constituted” by episodes of time-consciousness and is correspondingly in a permanent state of flux by no means implies that the respective moments of time form an “A-series” in McTaggart’s sense of the term, i.e., that as a result of their “changeableness” with respect to future, present and past they build a temporal structure different from the series of intrinsically unchangeable, objective moments of time.^33^ It merely implies that the temporal stages of movement are *perceived* by us under different *indexical aspects* which succeed each other according to a rule that is constitutive for our *time-consciousness*: namely future, present and past. The notion of an A-series is therefore not required in order to take into account the dynamic nature of the according temporal determinations. We are not dealing with intrinsic properties of the corresponding temporal phases here but rather with relational properties of these phases, properties that they merely possess with respect to the mental episodes in which they “constitute themselves,” i.e., in which they present themselves to our consciousness.

Taken in this generality, though, the analogy between intentional consciousness and the perception of movement fails to deal with the other indexical aspect (besides the concept of the present) of the content of the meta-belief whose (factual or counterfactual) presence the metadoxastic view regards as the characteristic mark of intentional consciousness. I mean the aspect expressed by the first-person pronoun “I” (and denoted by the corresponding quasi-indicator). In order to produce an analogy that captures this “I”-aspect, too, I propose to construe conscious intentional states after the model of propriophysical perceptions *of one’s own bodily movements*: For instance, just as I see that *my own* hand is *now* moving, so I believe that *I now* believe that it’s raining when I judge, i.e., *consciously* believe: “It’s raining.”

This analogy highlights the dynamic nature of intentional experiences, i.e., the fact that they are always embedded in transtemporal, variable cognitive structures in which a particular object (such as the state of affairs that it’s raining at a certain time and place) is continuously represented (if only implicitly) as identical throughout a period of time. It is indexical time-consciousness that makes synthetic cognitive achievements (*kognitive Syntheseleistungen*) such as this possible.

But does this consideration not make it clear that the indexical metadoxasitic view is *circular* [objection #4]? No. True, it demonstrates that according to that view, intentional consciousness *presupposes* time-consciousness. However, this is a thesis about an existential dependence relation that does not at all imply that the *concept* of intentional consciousness contains the *concept* of time-consciousness. The dispositionalist higher-order theory merely claims that the indexical concept of *now* is involved in the contents of the meta-beliefs forming the top of the structure of intentional consciousness; that this concept is applicable on the basis of certain sorts of intentional experience only is quite a different matter.

There is a further respect in which propriophysical perception can serve us as a model for intentional consciousness. Like the objects of the required meta-beliefs, the objects of these perceptions are subject, at least in principle, to the respective subject’s *rational self-control.* For where some of our bodily movements (such as someone’s “instinctively” opening his or her hands after having grasped a hot potato) occur rather involuntarily, we do seem to have the freedom, at least principally, to refrain from performing an action, and we are capable, it seems, to critically reflect upon the intentions underlying our actions, so as to make responsible practical decisions. Something similar goes with regard to intentional experiences. They, too, sometimes occur quite involuntarily, as when a particular thought suddenly crops up; but in principle we can make them subject to our rational control. As John McDowell has pointed out, this even holds true of those “receptive” sense-experiences on which our singular perceptual judgments (such as “This is red”) are based:

“How one’s experience represents things to be is not under one’s control, but it is up to one whether one accepts the appearance or rejects it.”^34^

“The point here is well illustrated by familiar illusions. In the Müller-Lyer illusion, one’s experience represents the two lines as being unequally long, but someone in the know will refrain from judging that that is how things are.”^35^

When we undergo a perceptual illusion, like the Müller-Lyer illusion, then *nolens volens* sense-experience presents a state of affairs to us that does not really obtain. However, it is open to us to call into question, in the light of our belief-system, the reliability of the experience in question – and, if necessary, to reject its content as inaccurate (non-veridical).^36^ As a propositional (hence, intentional) experience it is accessible to rational self-control.

Now (executed) rational self-control requires *self-consciousness* (introspective or reflexive awareness). If an intentional state is to be open to critical reflection, it is not enough that this state be conscious (which would require no more than unconscious indexical meta-beliefs). Rather, its subject must in addition be *aware of this state as belonging to himself* (and being subject to his control). In other words: the respective state of consciousness must be represented by a *conscious* meta-belief.^37^ According to the indexical metadoxastic view this means that the subject must have (at least) a third-order belief (“I now believe that I am in such-and-such an intentional state now”).^38^ It is a characteristic feature of conscious intentional states that they are introspectively available in this way to their subject. To this end, the respective subject must, however, already possess the required mental (folk-psychological) concepts, such as the concept of belief, and he must be able to consciously apply them to himself. Therefore, small children often do not yet have self-consciousness at their disposal, so that they are still unable to critically reflect upon their intentional experiences. It is for this reason that they do not yet count as fully responsible moral persons.

It is often objected to metarepresentational theories that they ignore a phenomenological datum which becomes quite evident in connection with self-consciousness, notably the *transparency* of consciousness [objection #5]. If you are asked to closely reflect upon the way you are appeared to, say, when you consciously see a house, then your whole attention will be focused on the intentional *object* of the relevant experience (and to its perceivable qualities), i.e., on a particular *house* rather than on your own *perception* of the house (or so it would seem). Hence the respective experience seems to be transparent as far as its introspectively accessible qualities are concerned:

“When you try to examine [your experience], you see right through it, as it were, to the qualities you were experiencing all along in being a subject of the experience, qualities your experience is *of.*”^39^

Given that even our introspective glance at a given conscious perception actually “goes trough” the perception and is directed at its object, the assumption that a given experience is conscious in virtue of a meta-representation of that experience simply appears to be mistaken; or so the objection goes.

Whatever the force of this objection when raised against other metarepresentational theories, it is ineffective against the indexical metadoxastic view. In order to be introspectively aware of, say, a chestnut, you must, on this view, believe that you now believe that you are seeing a particular chestnut. How does this third-order belief emerge? In the veridical case (in which there really is a chestnut tree in the perceptual field) a crucial part of the answer is that you *direct your conscious attention to the chestnut-tree in your perceptual field* – this will enable you to describe the tree *as it perceptually appears to you*: “I now see this tree as having green leaves, as blooming, with white blossoms...” This description expresses, as its condition of sincerity, a second-order belief. Why is it supposed to be that implausible to hold that it even expresses a third-order belief in the case of introspective report? At any event, the indexical metadoxastic view seems to be compatible with the phenomenon referred to as the transparency of consciousness.

The claim that the meta-beliefs normally required for intentional consciousness are *indexical* in character enables me to meet an obvious objection that Robert Kirk has raised against metadoxastic, or dispositionalist higher-order order judgment, theories of consciousness [objection #6]:

“An obvious difficulty is that although all sorts of things are *available* to be thought about – holiday memories, for example – most of them are not conscious.”^40^

My reply is that while it *is* of course possible to have, say, certain holiday memories without consciously revel in those memories, the fact remains that in a case such as this the content of the relevant meta-belief lacks a certain indexical element: one fails to take oneself to remember such-and-such holiday experiences *now*. The experiences in question are, in other words, not *present* in memory. I thus conclude that Kirk’s objection does not affect the *indexical* metadoxastic view.

A related objection [objection #7] is driving at the *dispositional* character of the relevant kind of meta-belief. According to the indexical metadoxastic view, I am in a *conscious* perceptual state if I am merely *disposed* to judge, on the basis of the perceptual state, that I am in that state now. But what about Armstrong’s absent-minded car-driver? Is he not disposed to make a judgment such as this as well? I think not. For how should the driver judge himself to perceive such-and-such *now* when he is half asleep? But if he is presently unable to *judge* himself to perceive the street now, as he seems to be, then he cannot be *disposed* to judge so *at this very time*, either. For, surely, you cannot be disposed to make some judgment at a particular time unless you are able to perform this judgment *at that time*. Consequently, the indexical version of the metadoxastic view is indeed able to explain the lack of intentional consciousness that almost proved to be disastrous for Armstrong’s absent-minded car-driver.

I have argued that intentional consciousness has the general form of the *cogito*, in that (to vary a relevant formulation by Kant) the “I am just undergoing such-and-such an experience” must be able to accompany all of my intentional experiences. As for the indexical content of the according meta-beliefs, I have so far only been concerned with its *temporal* aspect, i.e., the concept of *now.* What about the aspect expressed by the first-person pronoun “I” and its quasi-indexical counterparts like “he himself,” i.e., the concept of the “self”?

I have already indicated that where I take some simultaneous conscious representation of *now* to be an indispensable precondition of any intentional experience, I do not think that something similar goes for *I*. After all, there are “self-forgetting” mental states, e.g., when someone’s thoughts are completely absorbed by a theoretical problem. According to the metadoxastic view, this means that no second-order belief concerning oneself *as referring to oneself* is generally required. The underlying assumption to the effect that our “I”-consciousness displays a conceptual meta-structure^41^ is quite plausible on independent grounds. Thus, if someone affirms “I have a broken leg,” then he *eo ipso* knows himself *to be referring to himself* by “I”; he could immediately add something like “I am talking of *myself*”.^42^ If, however, someone refers to himself by a singular term which does *not* express the “I”-concept, then it is by no means guaranteed that he refers to himself *knowingly*. Think of a speaker who unknowingly sees himself in the mirror and declares “*That person* has a broken leg.”

But what about “identity disorders” such as Cotard’s syndrome or reverse intermetamorphosis, where subjects deny their own existence or firmly believe themselves to have become someone else [objection #8]?^43^ Do these subject not lack the relevant meta-belief (“I am thinking of myself”), despite the fact that they consciously refer to themselves when they make a judgment such as “I am dead” or “I am Douglas”? I think not. These subjects would not at all deny – in fact: they would (be disposed to) affirm – that they are referring to themselves when claiming that they are dead or, say, Dougie. Their pathological beliefs about themselves are certainly abnormal and do not qualify as cases of self-*knowledge*; but they still display a subjective perspective that cannot be consciously executed absent a meta-perspective representing oneself as currently referring to oneself. The fact that some Cotard patients even stop using the pronoun “I” and replace it by a proper name is no disproof. But even if these patients were completely unable to take a first-person perspective (which I regard as controversial), so that they lacked the capacity to be in states of “I”-consciousness, the following kind of counterfactual would still hold true for them, because they represent abnormal cases: if the respective subject had the “I”-concept in his repertoire, he would be disposed to judge that he thinks of himself (namely, as being in such-and-such a mental state) whenever he undergoes an intentional experience. And this is all that the indexical metadoxastic view actually requires.

## 4. The Unity of Consciousness

A related field of application of this theory is the twofold problem of how to construe (1) the synchronic and (2) the diachronic *unity of consciousness*, respectively.

Let me begin with question (1):

Which conditions must obtain in order for two *simultaneous* intentional experiences to belong to the same consciousness?

To answer this question, it is useful to consider the phenomenon of temporary *splits* of consciousness, i.e., cases of temporary *reduplication* of a subject’s synchronic unity of consciousness. Inspired by psychological experiments where the upper brain hemispheres of epileptic patients have been separated to generate “two seperate spheres of consciousness” or “streams of consciousness” (Sperry),^44^ Derek Parfit has described a fictitious example that seems to be particularly apt to illustrate the phenomenon in question.^45^ Here it is:

The right brain hemisphere is (roughly speaking) responsible both for the representation of our left visual field and for the execution of our left hand’s movements, whilst the left hemisphere is responsible for the right visual field and the movements of the right hand. Now let’s assume that I am taking a written examination in physics. I only have 15 min left to solve a difficult problem. I have in mind two different (and clearly incompatible) solutions. I do not know which one yields the correct answer. Fortunately, scientists have provided me with a device that enables me to separate my two hemispheres by lifting my eyebrows until I raise my eyebrows again. This enables me to develop both of the two solutions for 10 min and to select the better solution during the last 5 min. The right hand writes down the solution developed in the left hemisphere, while the left hand writes down the solution developed in the right hemisphere. Meanwhile, I observe, in my right visual field, my left hand writing down a solution *without* at the same time being aware, in the left hemisphere, of the corresponding experiences (of calculating and writing) that occur in the right hemisphere. (The same goes, *mutatis mutandis*, for the other hemisphere.) After 10 min I reconnect the two hemispheres. My two streams of consciousness of the last 10 min flow together again and I can suddenly recall the experiences from both hemispheres.

The (successful) experiments which inspired Parfit to imagine this case support the assumption that this kind of scenario is not as unthinkable as one might suspect at first glance. The point of the example is that sameness of bodily subject (here: of the examinee) undergoing a number of experiences (here: calculation and conscious writing) does not imply sameness of the streams of consciousness to which the relevant experiences belong. Accordingly, a person’s conscious life sometimes more resembles (to use a nice metaphor by Parfit) a river occasionally dividing up into separate streams rather than a channel.^46^

Now let us assume that I realize that I am making two judgments: I am judging both that *p* and that *q*. Then my two judgments will automatically belong to one and the same stream of consciousness. Thus, to modify the above example, if I had noticed 14 min before the end of Parfit’s physics exam that I am currently judging *p* in connection with solution A and *not-p* in connection with solution B, then my attempt at separating my consciousness into two streams by lifting my eyebrows would have failed 1 min before. Parfit therefore proposes the following theory of the unity of consciousness at a time:

“[…] what unites my experiences in my right-handed stream is that that there is, at any time, a single state of awareness of these various experiences […] At the same time, there is another state of awareness of the various experiences in my left-handed stream. My mind is divided because there is no single state of awareness of both of these sets of experiences.”^47^

To put the same point in a positive way:

Actualist higher-order judgment theory of the synchronic unity of consciousness

Two simultaneous intentional experiences belong to the same stream of consciousness iff they are both intentional objects of one and they same meta-judgment of the sort “I am now having such-and-such experiences.”

However, this view seems to lead into malicious regress once again, for the relevant meta-judgment must surely itself belong to a stream of consciousness, and presumably to the same one as the experiences it is about; so it must again be the object of a higher-order judgment, and so on, *ad infinitum.*

The indexical metadoxastic view avoids this regress by merely requiring the presence of a suitable “apperceptive” meta-*belief*, i.e., a corresponding judgmental *disposition*:

Dispositionalist indexical higher-order judgment theory of the synchronic unity of consciousness

Two simultaneous intentional experiences belong to the same stream of consciousness iff they are both intentional objects of a meta-belief of the sort “I am now having such-and-such experiences” that would be manifested (actualized) by one and the same meta-judgment (where the temporal demonstrative specifically refers to the moment of time at which both of these experiences occur).

The relevant meta-judgmental disposition is founded in, and motivated by, internal-time consciousness in which the “absolute simultaneity” (Husserl) of the lower-order experiences in question is sensed, i.e., their identical position in the subjective flow of time. According to the corresponding metadoxastic view, the synchronic unity of consciousness is not transitive. For instance, from the fact that the simultaneous experiences *e_1_* and *e_2_, e_2_* and *e_3_* as well as *e_3_* and *e_4_* belong to the same consciousness *in pairs* it does not follow that, e.g., the experiences *e_1_* and *e_4_* belong to the same consciousness. For, why should it be impossible that the subject who undergoes *e_1_* through *e_4_* believes of (1) *e_1_* and *e_2_*, (2) *e_2_* and *e_3_* and (3) *e_3_* and *e_4_*, respectively, that he is now having those experiences, *without* at the same time being disposed (4) to make a corresponding meta-judgment about *e_1_* and *e_4_*?^48^ The answer “Well, for the simple reason that under these conditions *e_1_, e_2_, e_3_*, and *e_4_* would not belong to the same consciousness” is not legitimated by the metadoxastic view. Hence, it seems to be possible for a subject to have two consciousnesses with overlapping experiences at the same time.^49^

I do not regard the abandonment of the principle of transitivity regarding the synchronic unity of consciousness as an undesired consequence. Quite on the contrary: in the light of more recent neurophysiological findings, philosophical thought-experiments can be construed that make this principle appear quite problematic.^50^

In the experiments I have in mind, split-brain patients are confronted, in their two visual fields, with different numerals, e.g., “6” and “7,” and are asked to successively answer, by means of their hands, first the question whether the corresponding numbers are *identical* and then the question which number is *higher* (i.e., greater) and *lower*, respectively. The patients managed to solve the second problem with ease but were incapable of solving the first one. This irritating result was confirmed by repeated performances of the experiment. Although the consequences of experimental findings such as this regarding the synchronic unity of *consciousness* are controversial^51^, they do give rise to indeed coherent thought-experiments such as the following.

Let us assume that the number in the patient’s left visual field is consciously perceived, in his right hemisphere, both as *a 6* and as *lower*, whereas the number in his right visual field is consciously perceived, in his left hemisphere, both as *higher* and as *a 7*, such that the following holds true: the first experience (the “6”-perception) belongs to the same consciousness as the second one (the “lower”-perception), the second experience belongs to the same consciousness as the third one (the “higher”-perception), which in turn belongs to the same consciousness as the fourth experience (the “7”-perception), but this last experience does *not* belong to the same consciousness as the first one. In this situation, synchronic unity of consciousness obtains between particular intentional experiences of the patient, yet if we take into account the whole of these experiences, transitivity does not obtain. We are dealing with a synchronic split of consciousness, such that the fourth experience does not belong to the same consciousness as the first one.

The metadoxastic view accounts for this possibility by its requirement that two experiences – in this case: two conscious perceptions of different numerals – do not belong to the same consciousness, at a given time, unless the subject is disposed, at that time, to judge himself to be undergoing both of these experiences. This requirement is violated in the present thought-experiment – which is just as it should be.

Let me now turn to the problem of the *diachronic* unity of consciousness, i.e., question (2):

Which conditions must obtain in order for two intentional experiences *occurring at different times* to belong to the same consciousness?

To answer this question, consider a modified version of the above example with the physics exam. Imagine that the device for re-uniting my two streams of consciousness has been irreparably destroyed, so that my consciousness turns out to be irrevocably divided into two separate streams 15 min before the end of the exam.

What is happening here? From the time of fission nobody will ever be able again to *remember*, from the first-person perspective, any pair of experiences from *both* of the two streams of consciousness at the same time. Not so in Parfit’s original version of the example: true, I cannot make a memory judgment of the type “I just (/earlier/once) had such-and-such experiences” about *both* a particular experience from the left hemisphere *and* a particular experience from the right one during the 10 min lasting split of consciousness; but *after that* I am – at least in principle – capable again of making an “apperceptive” memory judgment such as this, provided I have an according meta-belief. The two streams of consciousness are thus re-united – *à la longue* they are part of the same river, to take up Parfit’s metaphor again. So *diachronically* speaking, the various experiences from these two streams do indeed belong to the unity of a single stream of consciousness. Thus we have:

Dispositionalist indexical higher-order judgment theory of the diachronic unity of consciousness

Two diachronic intentional experiences belong to the same stream of consciousness iff both of them are intentional objects of a meta-belief of the sort “I just had such-and-such experiences” or “I earlier had such-and-such experiences” that would be manifested (actualized) by one and the same meta-judgment.

Again, the relevant meta-belief is founded in, and motivated by, internal time-consciousness. There is a continuous chain of retentions tacitly leading back to the experiences that can be self-ascribed in a corresponding “apperceptive” memory judgment:

“[…] notably, when from it [i.e., the recollection in question, or rather its intentional object; CB], we get, through a continuous path of memory, to the ‘now’ and then again from the ‘now’ through retention, i.e., continuously reviving retention, back to what is posited in the recollection.”^52^

A striking consequence of this metadoxastic view is that, just like the unity of consciousness at a time, its unity across time fails to be transitive. For, let *x* be a stream of consciousness irrevocably dividing, at a time *t*, into two seperate streams *y* and *z*. Let *e_1_* be an (intentional) experience occurring in *x* before *t*, let *e_2_* be an experience occurring in *y* after *t* and *e_3_* an experience occurring in *z* after *t*. Let us further assume, first, that at some time somebody, call him *S_1_*, remembers *e_1_* and *e_2_* from the first-person perspective and, secondly, that at some time someone, call him *S_2_*, remembers *e_1_* and *e_3_* from his first-person perspective.^53^ Then *e_2_* will be part of the same stream of consciousness as *e_1_*, the same goes for *e_3_*, but *e_2_* and *e_3_* will nevertheless belong to different streams of consciousness. Or so the present theory has it. To my mind, this result is, once again, just as it should be. For, it reflects that fact that *S_1_* and *S_2_* are, arguably, two different descendants of a person that ceased to exist at *t*.

Instead of going into the vexed philosophical problem of personal identity further, let me end by highlighting another merit of the present view, one that is more relevant to the topic of this paper. It concerns the phenomenological deep- or microstructure of intentional experiences purporting to represent spatio-temporal particulars. The present theory of the unity of consciousness manages to shed some further light on this structure, in addition to what has already been said about this structure in section 1. As an example, consider again the case of a continuous series of (conscious) perceptions the whole of which constitutes the perception of a physical movement (motion). Husserl has pointed out that a complex perception such as this has an underlying microstructure of conscious states, which structure he describes as internal time-consciousness:

“Primary memory, or as we said, retention, continuously attaches itself to the ‘impression.’ […] In the case of the perception of a temporal object […], the perception terminates at any moment in a now-apprehension […]. During the time that a motion is being perceived, a grasping-as-now takes place moment by moment; and in this grasping, the actually present phase of the motion itself becomes constituted. But this now-apprehension is, as it were, the head attached to the comet’s tail of retentions relating to the earlier now-points of the motion […] Thus a pushing back into the past continually occurs. The same continuous complex incessantly undergoes a modification until it disappears; for a weakening, which finally ends in imperceptibility, goes hand in hand with the modification.”^54^

For the sake of simplicity, let us assume that the perception of motion consists of three successive partial perceptions, *P_1_, P_2_* and *P_3_*, of a moving object *x*. As regards the experiences *P_1_-P_3_*, this is surely a paradigm case of diachronic unit of consciousness. Now we can apply the metadoxastic theory to this example by describing it with recourse to appropriate “apperceptive” judgmental dispositions. If we do so, we obtain what seems to me to be an illuminating analysis of the structure of consciousness at issue, as follows:

At time *t_1_, P_1_* is founded in – is existentially dependent upon – both an underlying awareness of the present and an underlying egocentric spatial awareness, such that the subject, *S*, who undergoes *P_1_* at *t_1_* is disposed to judge, about *x*:

*t_1_* “I am *now* seeing *this here*” (where “this” refers to the moving object *x* and “here” refers to the place such that the subject *S* sees *x* to be located at that place, from his or her egocentric perspective, at *t_1_*).

At *t_2_, S* still immediately remembers *P_1_*, such that *S* is disposed, on the basis of both this retentional awareness and *P_2_*, to make the following judgment about *x*:

*t_2_* “I saw this *over there just a moment ago* and I am *now* seeing it *here*” (where the spatial demonstrative “over there” reflects *S’* altered egocentric perspective upon *x* at *t_2_*).

Finally, at *t_3_, P_1_* appears more distant (“pushed back”) temporally (*S*’ memory of *P_1_* begins to fade); at the same time, *S* immediately remembers *P_2_*, so that *S* is disposed, on the basis of both of these memories and of *P_3_*, to judge about *x*:

*t_3_* “I *earlier* saw this *over there*, while I was seeing it *over there just a moment ago*, and I am *now* seeing it *here*”.

The intentional contents of the described meta-judgments are obviously interrelated, according to a rule of temporal-indexical representation, such that the different temporal demonstratives (“now,” “just a moment ago” and “earlier”) express the dynamic egocentric aspects of the present moment, the immediate past and the earlier past, respectively. Thus, the continual “pushing back” of the earlier “now-points of the motion” into the past can be explained by recourse to the constant adaptation of the relevant temporal-indexical concepts (*now, just a moment ago, earlier*) to *S’* altered cognitive perspective upon *x* at *t_1_* and *t_2_*, respectively (according to a rule that is constitutive for the proper application of these concepts).

I thus conclude that the indexical metadoxastic view throws light on the structure of a number of phenomena that an adequate theory of subjectivity must not leave out of account: “I”-consciousness, synchronic as well as diachronic unity of consciousness, and time-consciousness. At the same time, Husserl’s conception of internal time-consciousness (conceived of as *pre*-pre-reflective self-awareness) helps explain what motivates the indexical meta-beliefs that this view of consciousness refers to.

## Author Contributions

The author declares that he is the sole author of this contribution.

## Conflict of Interest Statement

The author declares that the research was conducted in the absence of any commercial or financial relationships that could be construed as a potential conflict of interest.

## Footnotes

1 Cf. Husserl, 1950-, Hua XIX/2, pp. 565, 573f, 675 ff and the interpretation of these and related passages in Beyer, 2000, 86–104, 108 ff.

2 Cf., e.g., Husserl, 1950-, Hua I, p. 82 (“Every experience has an […] intentional horizon of reference to potentialities of consciousness […]”) and the interpretation in Smith and McIntyre, 1982, 239–264 (keyword: act-horizon).

3 Cf. Rosenthal, 1993, 204f.

4 Cf. Husserl, 1950-, Hua VIII, p. 90.

5 Cf. Husserl, 1950-, Hua VIII, p. 90.

6 Cf. Zahavi, 2002.

7 Cf. Beyer, 2011.

8 See Walton, 2003.

9 Cf. Zahavi, 2015, p. 180.

10 Cf. Zahavi, 2015, p. 180; emphasis mine.

11 Cf. Beyer, 2011, p. 44.

12 Cf. Zahavi, 2015, pp. 178, 181.

13 Cf. Husserl, 1950-, Hua III/1, p. 87; Husserl, 1950-, Hua XVII, p. 279.

14 Cf. Husserl, 1950-, Hua XX/1, p. 178; Husserl, 1950-, Hua IV, p. 222.

15 Cf. Husserl, 1950-, Hua XXXVI, p. 139.

16 Cf. Zahavi, 2015, 180f.

17 Also see the critical discussion of Zahavi’s interpretation of Husserl’s notion of internal time-consciousness in terms of “self-manifestation” in Beyer, 2011, p. 49.

18 Cf. Zahavi, 2015, p. 181.

19 Cf. Husserl, 1950-, Hua III/1, pp. 295f.

20 “He (she) himself (herself)” is to be interpreted as a quasi-indicator.

21 Cf. Armstrong, 1968, 93f.

22 Armstrong seems to hold a similar view: see Armstrong, 1968, 95 in the light of pp. 208–244 and especially p. 222.

23 In this article I do not argue for the underlying assumption that, at least in the case of normal adults, judgments (and other intentional experiences possessing propositional content) take place in the medium of intuitively (e.g. auditively) represented natural language sentences.

24 Cf. Davidson, 2001, 308.

25 We owe these experimental findings to the psychologist Josef Perner and his collaborators. Cf. Perner et al., 1987; also see Botterill and Carruthers, 1999, 93f. More recent findings suggest, though, that this concept of belief may be regularly acquired by children much earlier. See Goldman, 2006, p. 77.

26 Cf. Dretske, 1995, 110f.

27 Cf. Beyer, 2000, 118f.

28 As soon as Anna actually has meta-beliefs such as this, she can e.g. intentionally pretend to be convinced that the weather is fine (despite the fact that she is well aware of the fact that the weather is nasty). In this case, the intend to defraud is based upon Anna’s conviction that she herself believes that it’s raining.

29 After developing this argument I noticed that Peter Carruthers ascribes a similar idea to his former PhD student Sarah Clegg; cf. Carruthers, 2005, sec. 2.

30 Cf. Carruthers, 2000, 227f.

31 This objection was raised, in similar terms, by an anonymous referee.

32 Cf. Husserl, 1980, p. 25; see also Dummett, 1978.

33 Cf. McTaggart, 1908.

34 Cf. McDowell, 1994, p. 11.

35 Cf. McDowell, 1994, p. 11, fn. 9.

36 In this case, we do not regard the experience as a perception but rather, more cautiously, as an *appearance*; where the content of the introspective awareness underlying our critical self-reflection (see the next paragraph) runs, e.g.: “The two lines *now look* unequally long *to me*” or, even more cautiously, “*It seems to me as if I were now seeing* two unequally long lines.”

37 Of course, this is compatible with the existence of (even a large amount of) experiences that “cannot be inhibited, suspended, or terminated,” such as episodes of “mind-wandering” (cf. Metzinger, 2015, 275).

38 A similar view on self-consciousness is held by Rosenthal; cf. Rosenthal, 1993, p. 210.

39 Cf. Tye, 1998, p. 653. See also Harman, 1997, esp. p. 668; Dretske, 1995, 62 f; Carruthers, 2000, p. 123, 184f, 238 ff.

40 Kirk, 2001, p. 160.

41 Cf., e.g., Husserl, 1984, p. 813.

42 Cf. Spitzley, 2000, p. 40.

43 Cf. Metzinger, 2003, pp. 452–461.

44 For a philosophical discussion of the relevant experiments that partly confirms Sperry’s characterization cf. Nagel, 1979, pp. 156–162.

45 For the following cf. Parfit, 1984, 245 ff.

46 Cf. Parfit, 1984, p. 247.

47 Cf. Parfit, 1984, p. 250.

48 I do not know how to reformulate the premiss that this is indeed possible in a truth-preserving way without invoking the notion of a meta-belief. I regard it as an advantage of the metadoxastic conception of the unity of consciousness that it allows us (by means of this premiss) to derive the consequence that the principle of transitivity regarding the synchronic unity of consciousness is false (see the following four paragraphs).

49 Thanks to an anonymous referee for pressing me on this point.

50 For the following paragraph cf. Hurley, 1994, 68 ff. We owe the relevant data to Justine Sergent.

51 Cf. Marcel, 1994.

52 Cf. Husserl, 1950-, Hua X, p. 345.

53 Strictly speaking, “memory” is to be understood here as denoting *quasi-memory*. Experiences of quasi-memory are phenomenologically indistinguishable from those conscious mental episodes that we ordinarily describe as experiences of memory, but unlike the latter they do not depend on personal identity through time: if A quasi-remembers an experience of B’s, then A and B are not necessarily the same subject.

54 Cf. Husserl, 1999, 190f.
